# The ever-expanding diversity and complexity of the *Arenaviridae* family

**DOI:** 10.1080/21505594.2023.2279353

**Published:** 2023-11-15

**Authors:** Hinh Ly

**Affiliations:** Department of Veterinary & Biomedical Sciences, College of Veterinary Medicine, University of Minnesota, Twin Cities, MN, USA

**Keywords:** *Arenaviridae*, antennavirus, hartmanivirus, innmovirus, mammarenavirus, reptarenavirus

Viruses are generally classified as DNA or RNA viruses based on the type of nucleic acids that make up their genomes. DNA viruses, in general, were phylogenetically more similar in genomic sequences and host specific than RNA viruses [1]. An example of RNA viruses with increasingly diverse genomic architectures and host species is the *Arenaviridae* family. This editorial article aims to highlight similarities and differences of known and newly discovered arenaviruses, their expanding host ranges, and the diseases that they can cause. As some of these viruses can cause severe and sometimes fatal diseases in humans, it is important to keep track of them to prevent potential future outbreaks.

The name arenavirus comes from the Latin word *arenosus* meaning “sandy” and *arena* meaning “sand,” in recognition of the “sandy” appearance of arenavirus particles under EM visualization conditions. Arenaviral genome typically consists of two and sometimes three single-stranded RNA segments called small (S), medium (M), and large (L). They are about 10.5 kb in length and encode viral proteins in each of the RNA segments in opposite polarities (i.e. ambisense orientation) that are separated by non-coding intergenic regions (IGRs). The smaller S and M RNA segments (2.0–3.5 kb) encode the nucleoprotein (NP) on the virus genome-complementary strand and the glycoprotein precursor (GPC) on either the virus genome-sense strand or genome-complementary strand. The larger L RNA segment (6.0–7.5 kb) encodes the Large (L) protein that consists of the RNA-dependent RNA polymerase (RdRP) and endoribonuclease domains on the virus genome-complementary strand and, in many cases, the zinc-containing (Z) matrix protein on the virus genome-sense strand (Figure 1). It is important to note that a unique feature that is shared among arenaviruses is that their mRNAs are capped but not polyadenylated.

**Figure 1.**
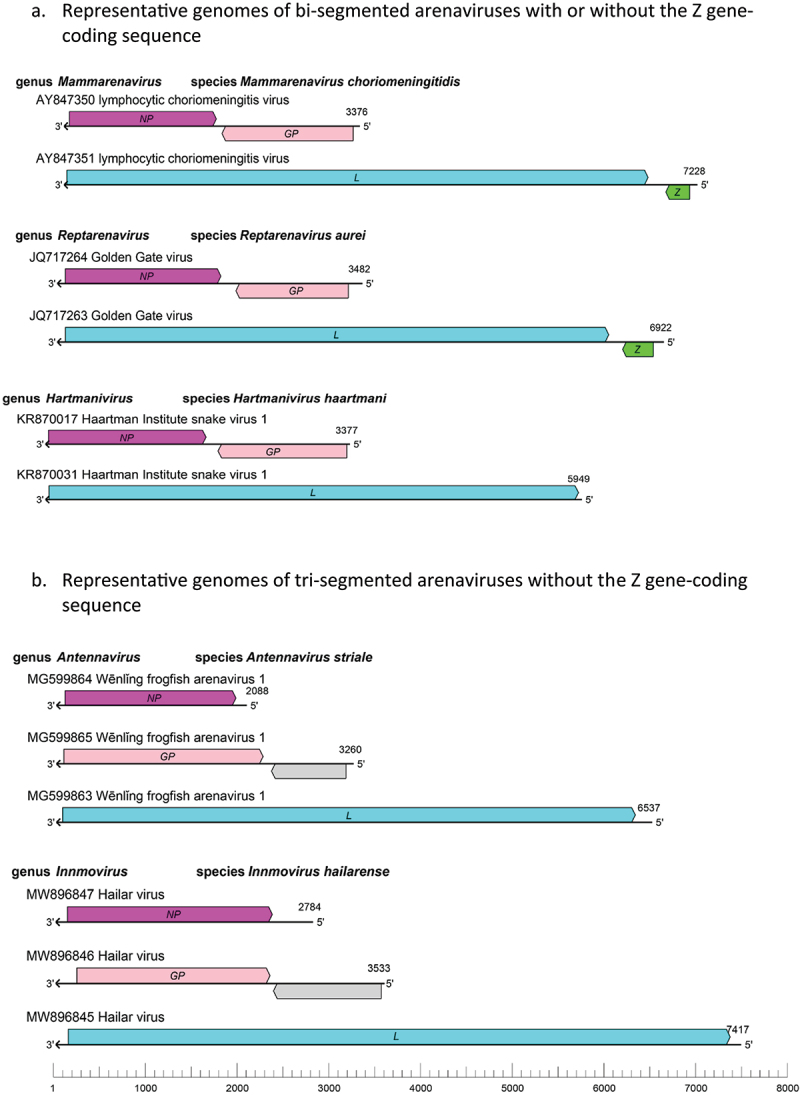
Schematic representations of bi-segmented (a) and tri-segmented (b) arenaviral genomes of the genus mammarenavirus (representative lymphocytic choriomeningitis virus with its genomic sequence designations AY847350 and AY847351 available in GenBank), genus reptarenavirus (representative Golden Gate virus with its genomic sequences JQ717264 and 717,263), genus hartmanivirus (representative Haartman institute snake virus 1 with its genomic sequences KR870017 and KR870031), genus antennavirus (representative Wenling frogfish arenavirus 1 with its genomic sequences MG599864, MG599865, and MG599863), and genus innmovirus (representative Hailar virus with its genomic sequences MW896847, MW896846, and MW896845). It is noteworthy that the genomes of the Haartman institute snake virus 1 (KR870031), Wenling frogfish arenavirus 1 (MG599863), and Hailar virus (MW896845) lack Z gene-coding sequences that are found in the genomes of the lymphocytic choriomeningitis virus (AY847351) and the Golden Gate virus (JQ717263). *GP*, glycoprotein gene; *L*, large protein gene; *NP*, nucleoprotein gene; *Z*, zinc-binding protein gene. Drawing adapted from that of reference #20.

The *Arenaviridae* family includes, at the time of this writing, 5 genera and about 60 known virus species. Some of these viruses infect fish (antennaviruses), snakes (reptarenaviruses and hartmaniviruses), mammals (mammarenaviruses), and yet other unknown hosts (innmoviruses). A recent taxonomic classification of these viral genera (www.ictv.global/report/arenaviridae) was based on analyses of complete *L* and *NP* nucleotide sequences and/or of the RdRP’s so-called “palm” domain sequences and was justified by the presence of broadly conserved domains within these proteins and by the rarity of RNA reassortment among the viral genomic segments. Taxonomic classification also took into consideration some phenotypic differences between these arenaviruses, such as their genome architectures, antigenicities, and ecologies.

Recent discoveries of some of these viruses suggest that they are ecologically diverse, and their geographic distributions tend to overlap with the distribution of the respective animal hosts that they infect. For example, reptarenaviruses and hartmaniviruses have been found to infect snakes [2–5]. The full natural host range of these reptilian arenaviruses is still unknown as they have only been isolated in captive snakes [2–5] and recently in indigenous captive and wild boid snakes in Costa Rica [6]. The Golden Gate virus (GGV), for example, was originally found (by metagenomic approach) in some boa constrictors, whereas the California Academy of Sciences virus (CASV) was found in annulated tree boas that were kept in captivity at the California Academy of Sciences, San Francisco, CA, USA [2]. Some of these reptarenaviruses can cause boid inclusion body disease (BIBD) naturally. However, a pure HISV-1 hartmaniviral isolate failed to induce any evidence of BIBD in experimentally infected cultured boid kidney cells prepared from a juvenile boa constrictor kidney [5].

Reptarenaviruses, whose name was derived from the Latin word *repere* meaning “creep” or “crawl” as a reference to the reptilian hosts of reptarenaviruses [7], contain a bi-segmented genome but are unique among known arenaviruses for their transmembrane surface glycoproteins (GP2) being more closely related to those of ebolaviruses (*Mononegavirales*: *Filoviridae*) than to those of antennaviruses, hartmaniviruses, mammarenaviruses, innmoviruses, or other bunyavirals. It is also interesting to note that, unlike other known cases of arenaviral infections, multiple reptarenaviruses appear to be able to infect the same snake as they often show multiple distinct small (S) and large (L) genomic RNA segments, suggesting either a high frequency of co-infections or that snakes can be infected with reptarenaviral particles that contain different combinations of the RNA segments. It is important to note that hartmaniviral genomes do not seem to encode for the Z protein that is found in the genome of mammarenaviruses and reptarenaviruses, the functional significance of which has not yet been demonstrated.

While some of the arenaviruses have been discovered in snakes (reptarenaviruses and hartmaniviruses), other arenaviruses have been found in invertebrates (e.g. Tacaribe virus TCRV in ticks) [8] and mammals [9]. Those that infect mammals are known as mammarenaviruses (from the Latin word *mamma*, which means “udder or breast” as they refer to the mammalian hosts of these viruses). Many of these viruses are known to infect rodents of one or a few species and are often geographically constrained to their natural rodent hosts, except for lymphocytic choriomeningitis virus (LCMV), which infects the ubiquitous house mouse (*Mus musculus*) and is therefore distributed worldwide. Other mammarenaviruses (e.g. TCRV) have also been found in Artibeus neotropical leaf-nosed, fruit-eating bats of the *Phyllostomidae* family [10]. The plateau pika viruses (PPVs) were found in small, mountain-dwelling mammals called pikas in Asia and Southeast Asia [11,12]. Other mammals, such as the Northern white-breasted hedgehogs in Hungary (Europe), serve as natural hosts for the Mecsek Mountains virus MEMV [13]. Other eulipotyphla, such as the Asian house shrews as well as eight other species of rodents in China were found to harbour the Wenzhou virus (WENV) [14], and three-toed jerboa (*Stylodipus sungorus*) found in the Inner Mongolia Autonomous Region (China) could play host to jerboa arenaviruses (JEAreV or Alxa virus) [15]. As such, natural host reservoirs and geographic distributions of mammarenaviruses appear to be quite diverse and complex.

While infections of their natural hosts are generally asymptomatic, zoonotic transmissions of some of the pathogenic mammarenaviruses into humans via contacts with infected animal’s carcasses or excretions or contaminated materials can lead to severe and sometimes fatal diseases with haemorrhagic or neurologic manifestations [16]. For example, the so-called Old-World (OW) mammarenaviruses [Lassa virus (LASV) and Lujo virus (LUJV)] and several New-World (NW) mammarenaviruses [e.g. Junin virus (JUNV), Machupo virus (MACV), Guanarito virus (GTOV), Sabia virus (SABV), and Chapare virus (CHAPV)] can cause endemic or sporadic outbreaks of haemorrhagic infections in West Africa and South America, respectively, whereas the OW mammarenavirus LCMV can cause encephalitic (neurological) disease in humans and can pose serious threats to the health of immunocompromised individuals that include but are not necessarily limited to pregnant people and those who receive solid organ transplants [9]. By some estimates, LASV alone is responsible for up to half a million of cases of infection and thousands of deaths in humans annually in several countries in West Africa and can be imported to developed countries via travel-associated means. It is thus essential to increase surveillance effort for these deadly mammarenaviral pathogens.

Using large-scale meta-transcriptomic approach, the authors of a recent study [17] screened 235 RNA-sequencing datasets from amphibian and reptile meta-transcriptomic data of 122 animal species covering 25 countries and identified 26 novel viruses that include a putative novel arenavirus (tentatively named Anole arenavirus) in the liver sample of *Anolis allogus*, which is a species of lizards found in Cuba. Similarly, analysis of a large-scale meta-transcriptomic dataset by other researchers revealed 214 vertebrate-associated viruses in reptiles, amphibians, lungfish, ray-finned fish, cartilaginous fish and jawless fish. Among those viruses are three species of the antennavirus genus of arenaviruses that consist of four viruses found in the actinopterygian fish [18]. The genomic sequences of the Wēnlǐng frogfish arenavirus 1 (WlFAV1) and Wēnlǐng frogfish arenavirus 2 (WlFAV2) were found in striated frogfish that do not appear to encode the Z protein. This coupled with the fact that the antennaviral genomes appear to consist of three RNA genomic segments, rather than two segments, is reminiscent of the genome architecture of the innmoviruses (Figure 1).

The genus of innmoviruses was recently established for a newly discovered single species of arenavirus discovered in river sediment samples without a known animal host. The innmovirus names were derived from Inner Mongolia Autonomous Region, China, where the Hailar virus (HLRV) was identified via genomic sequencing [19,20]. The fact that the innmovirus genomes appear to consist of three (S, M, and L) rather than two (S and L) genomic RNA segments and do not appear to encode a Z protein (Figure 1) suggests that the natural hosts of innmoviruses might be some fish species.

Whereas many of arenaviruses induce a persistent and frequently asymptomatic infection in the reservoir hosts, some of them can cause disease (e.g. boid inclusion body disease BIBD in some species of snakes) and zoonotic infections that can result in severe and sometimes fatal diseases in humans. As such, it is imperative that more research must be done to understand this family of interesting RNA viruses to prevent inadvertent importation of some of these viruses into non-endemic regions and to increase public-health safety.

## Data Availability

No primary data is included in this article.
