# Correction

**DOI:** 10.1080/21505594.2023.2287898

**Published:** 2023-11-27

**Article title**: The ever-expanding diversity and complexity of the Arenaviridae family

**Author name**: Hinh Ly

**Journal**: VIRULENCE

**Bibliometrics**: VOL. 14, NO. 1, 1-4

**DOI**: https://doi.org/10.1080/21505594.2023.2279353

When the article was originally published the Accepted date in the history date was present wrong as **Accepted 30 March 2023**. This change has now been rectified as **Accepted 30 October 2023** within the article, and the corrected article has been republished now.

